# Well Done, Montana!

**Published:** 1897-03

**Authors:** 


### WELL DONE, MONTANA!

ON other pages of this issue will be found two laws, one of which has passed, and the second will, in all probability. And when one reads them he will marvel at so great an achievement even in that hustling, up-to-date State. Many who had the pleasure of being with us at Buffalo will recall the presence of one of our older members whose face had not been seen in Association circles for several years, but when we recall his keen interest in all the proceedings at the "Queen City of the Lakes," we can say, with one accord, it was doubly good to have been there and to know now that from that convention there returned to Montana on the North our esteemed colleague, Dr. Knowles, imbued with the one strong thought that Montana should no longer bring up the rear guard. In the language of that hustling commonwealth, our colleague went up to Helena and camped on the battle-ground of legislative power, where he won for his profession, our cause, and the people of his State the powerful and valued influences of Senator Metzell, and two bills were prepared looking to a better inspection of food-products and safeguards for the veterinarians. Imbued with the necessities of this first act for a purer food-supply, realizing how closely linked this act must of necessity be with an educated and qualified veterinary profession, Senator Metzell sounded through the press of Montana, in vigorous and forceful language, the need of these legislative provisions, and when urging their adoption on the floor of the Senate he exhibited a fund of information on the subject of meat- and milk-inspection and a knowledge of the requirements of a good veterinarian that were refreshing to read. With energy and determination he pressed these measures so forcibly before his people that in a comparatively short period he secured the enactment of the pure-food law, and the other is in course of passage. We are glad and proud to have the privilege and honor of extending, on behalf of the veterinary profession of the whole United States, our sincere thanks and unmeasured appreciation to Senator Metzell for his earnest and successful efforts in our interest, and we look forward with confidence that, when the full value of these laws is realized by the people of Montana, they will find a still broader and higher plane of usefulness and power for Senator Metzell.

